# Whole person HIV services: a social science approach

**DOI:** 10.1097/COH.0000000000000773

**Published:** 2022-11-22

**Authors:** Alastair Van Heerden, Hilton Humphries, Elvin Geng

**Affiliations:** a Centre for Community Based Research, Human Sciences Research Council, Pietermaritzburg; b SAMRC/Wits Developmental Pathways for Health Research Unit, University of the Witwatersrand, Johannesburg; c Department of Psychology, School of Applied Human Sciences, University of KwaZulu-Natal, Pietermaritzburg, South Africa; d Centre for Dissemination and Implementation, Institute of Public Health, Division of Infectious Diseases, Department of Medicine, School of Medicine at Washington University in St. Louis, St. Louis, Missouri, USA

**Keywords:** HIV prevention, HIV treatment, human behaviour, implementation science

## Abstract

Globally, approximately 38.4 million people who are navigating complex lives, are also living with HIV, while HIV incident cases remain high. To improve the effectiveness of HIV prevention and treatment service implementation, we need to understand what drives human behaviour and decision-making around HIV service use. This review highlights current thinking in the social sciences, emphasizing how understanding human behaviour can be leveraged to improve HIV service delivery.

**Recent findings:**

The social sciences offer rich methodologies and theoretical frameworks for investigating how factors synergize to influence human behaviour and decision-making. Social–ecological models, such as the Behavioural Drivers Model (BDM), help us conceptualize and investigate the complexity of people's lives. Multistate and group-based trajectory modelling are useful tools for investigating the longitudinal nature of peoples HIV journeys. Successful HIV responses need to leverage social science approaches to design effective, efficient, and high-quality programmes.

**Summary:**

To improve our HIV response, implementation scientists, interventionists, and public health officials must respond to the context in which people make decisions about their health. Translating biomedical efficacy into real-world effectiveness is not simply finding a way around contextual barriers but rather engaging with the social context in which communities use HIV services.

## INTRODUCTION

The public health needs of the global HIV response are changing and, therefore, so must the scientific methods, perspectives and tools to stay abreast of these evolving needs. In 2022, 9 out of 10 women and 8 out of 10 men living with HIV are aware of their status yet between 20 and 30% have not yet initiated treatment [1,2]. The salient challenges are increasingly on the ‘right’ side of the cascade – with treatment initiation, and even more so, with retention and long-term engagement in care. Although these challenges have always been present, public health must now move more than ever from episodic interventions to sustainable engagement; from meeting a person's needs at particular moments to meeting them over decades; from understanding episodic behaviour to a wider and longitudinal understanding of the whole person in economic, social and organizational contexts. 

**Box 1:**
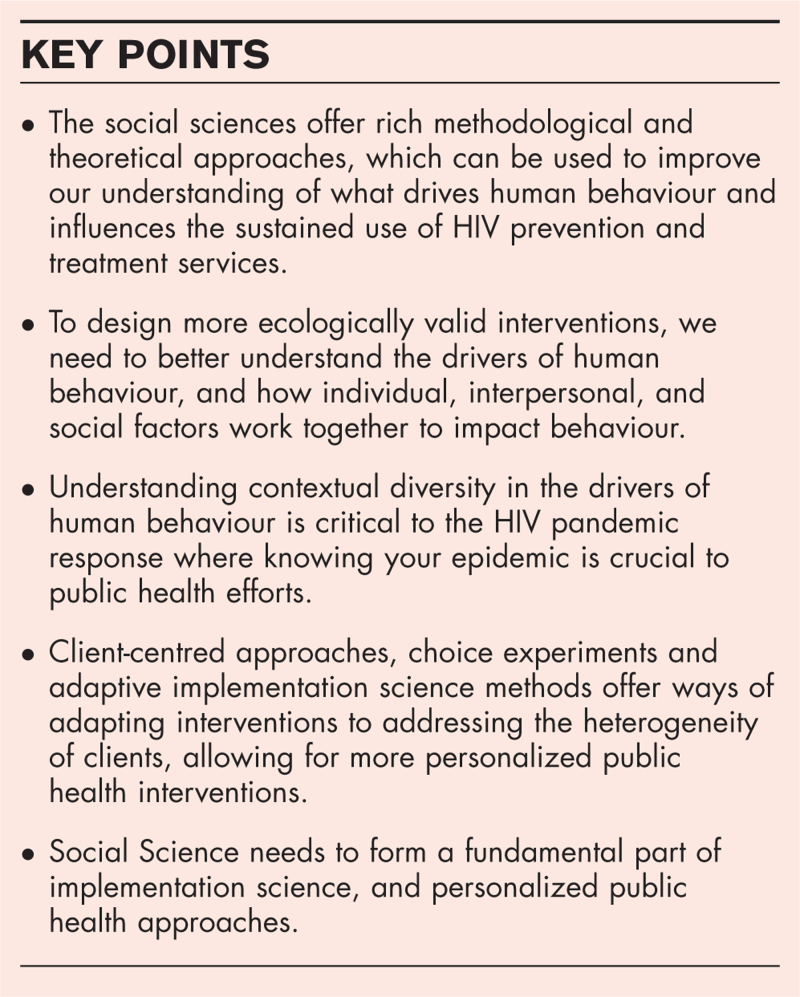
no caption available

The rising prominence of implementation research in the HIV field in part meets these needs by broadening our field of inquiry. For example, implementation science has asked us to direct our attention upstream of ‘adherence’ and individual level, psychological theories (e.g. information-motivation-behaviour [3]). Instead, we must ask about what opportunities and capacities exist for people to access treatment [4]; how to understand both beliefs and behaviour in the setting of social relationships (drawing from sociology); improving organizational behaviour (e.g. using organizational culture and climate [5]), and how organizations adapt and adopt new practices (e.g. using Normalization Process Theory [6]); or how to conceive of strategies to underlying implementation problems (e.g. using Behavioural Chance Techniques Taxonomy [7]).

In this manuscript, we suggest that one further framing that could magnify these diverse and broad perspectives from implementation science is the notion of the patient journey. The patient journey draws from the customer experience and customer journey, ideas that emerged from marketing in which users of a service are thought of through a series of touch points with a service, each of which has a set of feelings, motivations, in both ‘vertical context with organizational, social and economic domains of life, but also ‘horizontal’ context of past experiences and future expectations. Together, the shift towards understanding care as a journey is partially reflected in differentiated service delivery (DSD) models, which emerged in 2016 when the WHO updated their guidelines on the use of antiretroviral therapy (ART). Instead of asking *who is eligible* and when should they be started on ART, the conversation moved to *how do we provide client-centred and high-quality care* to all PLHIV while at the same time remaining cognisant of not placing additional burdens on the health system [8]. A longitudinal, multidimensional heuristic, centred on patient experience, can help advance our public health response.

Novel multistate and group-based trajectory modelling are one way to capture these care journeys [9^▪^]. These techniques embrace the fluid longitudinal nature of people's HIV journeys and provide more insight into how the ups and downs of people's lives impact their engagement with HIV services [10]. Programs designed with DSD in mind take advantage of the benefits associated with patient-centred care. As was evident at the start of the coronavirus disease 2019 (COVID-19) pandemic when no medical interventions existed, beyond a cure for HIV, ongoing engagement between those seeking preexposure prophylaxis (PrEP) or PLHIV and the health system will continue to shift from primarily a biomedical to an increasingly behavioural issue with implementation science playing a crucial role in building both demand-side and supply-side dimensions [11^▪▪^]. In this article, we will explore social science approaches to whole person care. We begin by describing the broad factors that influence human behaviour, move on to present current multidisciplinary efforts that aim to deepen our understanding of human behaviour, and finally present implications and insights of these theories as applied towards achieving the 95–95–95 targets.

## DESCRIBING CONTEXTS THAT IMPACT HUMAN BEHAVIOUR

People's engagement with HIV prevention and treatment services are often conceptualized as a linear cascade, but this does not adequately reflect that accessing and sustaining HIV service use often happens in a nonlinear manner, people's HIV journeys often involve cycling in and out of these services based on life circumstances [12]. These journeys are determined not only by individual factors such as genetics and lifestyle but also social circumstances, environmental affordances, and general socio-economic, cultural, and systems conditions [13]. Behavioural change frameworks are the bedrock of the socio-behavioural sciences and make it possible for complex theories about how people think and act to be applied in real-world contexts.

**FIGURE 1:**
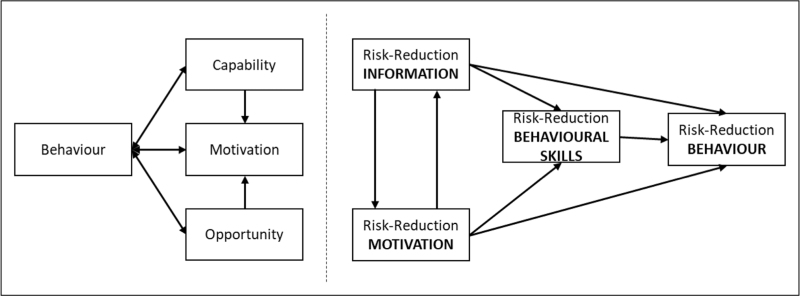
The COM-B model underpinning the behaviour change wheel (left) and the Information-Motivation-Behaviour Model (right).

Over the past decade, one of the most popular individual level models for health behaviour change has been the behaviour change wheel [14]. It suggests that all health interventions seeking to change behaviour need to take into consideration three critical conditions (Fig. 1, left): physical and psychological capabilities; motivation, which includes both conscious processes such as decision-making, and automatic processes including emotions and habits and the opportunities that exist beyond the individual that make the behaviour possible. The capability, opportunity, motivation, and behavior (COM-B) model sits at the centre of the behaviour change wheel with outer layers reinforcing the fact that interventions implemented to change individual behaviour, need to be cognisant of environmental, social, and systems factors within which the individual is operating. Alongside the COM-B model, other individual-level models that have been applied to HIV prevention and treatment interventions, including the Information-Motivation-Behavioural (IMB) skills Model, Health Belief Model, Social Cognitive Theory, the Theory of Reasoned Action, and the Stages of Change Model. Focusing on the IMB (Fig. 1, right) [3].

Informed by models like the behaviour change wheel, behavioural, environmental, social, and system interventions (BESSI) move beyond the individual behaviour to consider the influence of the social determinants of health (SDoH), as proposed by the WHO [14]. Although the SDoH is critiqued in the literature, the underlying principals espoused by the theory – that it is often the policies, systems and socio-economic circumstances of individual that drives their behavioural decision making – should be preserved [15,16]. Evidence is particularly robust for the fundamental role that socioeconomic factors such as income, wealth and education play in a wide range of health outcomes [17]. In response to a recent Lancet series on HIV in the USA, it was noted that the current USA HIV epicentre is in the 17 southern states that are characterized as having poorer health infrastructure, lower taxation, high stigma and fewer HIV care providers than in the north of the country [18]. Using annually collected cross-sectional data from over 15 000 PLHIV in the United States, the study found that as the number of SDOH indicators increased so did the likelihood of a PLHIV [19]. Inclusive approaches that understand behaviour within a broader SDOH framework would benefit from important formative research that focuses on better understanding the context in which interventions (research and public health) are implemented. This would enable an understanding of the important drivers of service use engagement that would transition research efficacy to effectiveness more effectively. Multilevel interventions are important, but must be contextually responsive and address contextually relevant drivers rather than broad, catch-all interventions.

## WHAT IS THE CURRENT INNOVATE IN THINKING ABOUT MULTIDISCIPLINARY APPROACHES TO HUMAN BEHAVIOUR

Expanding our understanding of SDoH is fundamental to our efforts to curb the HIV pandemic [1]. To maximize the effectiveness of the ever-expanding toolbox of prevention and treatment technologies, we need to better understand what drives human behaviour and decision-making, remaining cognisant that these processes happen within a social world. This requires building bridges between different but complementary disciplines within the natural and social sciences, incorporating insights and novel methods to design interventions that are more ecologically valid and better able to respond to the diversity and complexity of human behaviour [20^▪▪^,^21^].

The theoretical position underpinning the design of many HIV interventions are often simplistic, focused on the individual, and often assuming people do not change their behaviour because they are unaware or lack information about how to do so [22^▪^]. Therefore, most interventions assume a person will behave in a way that benefits them, making rational choices when provided with information from an expert, and ease of access to HIV services. Although these assumptions are relevant to explain certain behaviours, they do not fully account for the dynamic interplay of individual, interpersonal, social, and structural factors that influence how and what decisions are made over the life course [20^▪▪^,^21^,22^▪^,^23^]. Social–ecological models, such as the Behavioural Drivers Model (BDM) move us towards capturing more of this complexity [22^▪^,^24^]. Informed by theories across multiple disciplines, the BDM incorporates multiple drivers of behaviour from across different social science disciplines in a simplified structure. As with broader socio-ecological approaches, the model recognizes that behaviours are a result of a complex interaction of multiple determinants with the lived context of the individual, and that which factors are important depending on the context and behaviour being investigated [24]. The BDM provides a pragmatic framework to investigate what combination of factors determine behaviours in different settings – recognizing that the rich diversity of behavioural drivers mean that a single behaviour may be affected by different motives, some directly related to the individual decision-maker, some to the important groups they belong to, and some to the broader structural context [20^▪▪^].

Understanding contextual diversity in the drivers of human behaviour is critical to the HIV pandemic response where ‘knowing your epidemic’ is crucial to our public health efforts [1]. While many new tools exist and more are being developed to prevent new HIV infections and to better treat HIV-positive people, helping implementing partners to translate research efficacy to public health effectiveness will require better understanding the contextually relevant drivers of user behaviour [21,25]. Further, it requires responding to a rapidly changing world and researchers becoming more multidisciplinary. Globalization, social media, technological advances are rapidly changing societies – influencing how people consume information, creating new global social norms and supporting rapid cultural evolution – all of which influence how people behave [20^▪▪^]. This means expanding our list of what factors determine HIV risk and treatment behaviours, re-defining the pathways through which they work and being cognisant of the dynamism of these pathways. Several innovative research areas expand the factors we need to consider when thinking about changing human behaviour: better understanding how cognitive biases and heuristics impact health decision-making (especially in the age of social media), the role of technology in how people consume information and interact with healthcare, the evolving dynamics of individual and collective identities – especially within ever-changing political environments, the importance of mental health and the socio-emotional context, the impacts of globalization on definitions of meta-norms and cultural evolution, the need to monitor and improve our profiling of risk behaviours, and the need for greater methodological innovation to improve ecological validity and embrace global contextual diversity [20^▪▪^,^21^,^26^,^27^].

## IMPLICATIONS FOR THE PREVENTION AND TREATMENT CASCADES

Improving the implementation of programs targeting both HIV prevention and treatment need to tread carefully when integrating one of the discussed behavioural models. For example, even interventions informed by ecological models can succumb to the temptations of returning to an individual focus, only acknowledging the role of the social world without interrogating it. This still places the individual decision-maker at the centre of their context, assuming that they will make a rational choice, informed by an expert, unless a ‘social barrier’ prevents them from doing so [22^▪^,^28^,^29^]. In unpublished communications with the first author, a woman recently questioned the value of PrEP stating in paraphrase ‘so I must take this pill if I don’t have HIV and I must take this other pill in I do have HIV. I do not understand why you are offering me this (PrEP) pill’. Her confusion exemplifies how people's perceptions and internal accounting do not necessarily add up to the rational decision we would like them to make. This also serves to possibly reduce potential client's engagement with HIV services – if a client struggles to fit HIV service use within their actual lived context, and the expert advice is unable to help them overcome these struggles, then client activation to use, and engage with these services may be less likely, short-term or inconsistent. Client-centred approaches, choice experiments and adaptive implementation science methods offer ways of adapting interventions to addressing the heterogeneity of clients, allowing for more personalized public health interventions [30]. This will be critical if we want to understand how people's lives impact their ability to use services, and how the social world interacts with the individual to co-create decision and behavioural pathways – forming different profiles of people to which interventions can be targeted [22^▪^,^30^,^31^]. Innovation in creating more personalized HIV services should be underpinned by high-quality qualitative research that ensures pathways to change are contextually appropriate and relevant to the groups to whom the service is being targeted [20^▪▪^]. Further, sustainable programmes of qualitative and epidemiological investigation and monitoring should be undertaken to remain responsive to changes in HIV service use, risk factors and determinants of human behaviours to ensure programmes remain responsive to evolving populations over time. The use of innovative modelling (e.g. multistate) techniques could personalize public health approaches by supporting the creation of user-centric profiles of care. Ongoing modelling of big data sets could also alert to changes in human behaviour, for example, SRH uptake patterns, and allow for rapid program re-design to respond to these changes.

Stepping further, public health should include an effort to advance social justice as part of an acknowledgment that it is fundamental to a complete state of health [20^▪▪^]. The field needs to escape the clinic and include in its mandate social protection aimed, for example, at gender norms and other structural impediments. Findings from the ‘cash plus care’ studies suggest that such approaches are both feasible and hold promise for HIV prevention among adolescents [32,33]. In 2016, there were already over one billion people in at least 130 countries receiving nonconditional cash payments with evidence for these programs including positive impacts on education, health, financial circumstances, employment and empowerment [34]. Significantly, a recent metanalysis of the impact of cash transfers on HIV prevention suggest that cash alone is not as effective as programs, which seek to simultaneously improve social support and mental health [35]. These programs show a future in which the health of an individual truly considers the whole person and the context in which they live.

## CONCLUSION

The social sciences offer implementers a rich and innovative array of methods and theories that help us understand the different factors that work together to influence how people act and make decisions. However, to sustain positive gains in the HIV prevention and treatment cycle, researchers, interventionists and public health officials need to better respond to the context in which people make decisions about their health. Translating biomedical efficacy into real-world effectiveness is not a matter of finding a way around contextual barriers but rather engaging with the social context in which communities use HIV services.

## Acknowledgements

*I would like to thank the HSRC Sweetwaters team for their tireless work delivering HIV interventions into the Greater Edendale Area of KwaZulu-Natal, South Africa. Their efforts inspired many of the ideas share in this article.*

### Financial support and sponsorship

*This work was sponsored in part by a generous grant by the Bill and Melinda Gates Foundation for a project titled – Better Information for Health in South Africa: Describing the impact of a nonlinear HIV treatment cascade on true patient outcomes (INV- 016742).*

### Conflicts of interest

*There are no conflicts of interest.*

## REFERENCES AND RECOMMENDED READING

Papers of particular interest, published within the annual period of review, have been highlighted as:

▪ of special interest
▪▪ of outstanding interest
